# The evolution of European economic institutions during the COVID‐19 crisis

**DOI:** 10.1002/epa2.1100

**Published:** 2020-11-18

**Authors:** Antoine Camous, Grégory Claeys

**Affiliations:** ^1^ Department of Economics University of Mannheim Mannheim Germany; ^2^ Bruegel and Conservatoire National des Arts et Métiers

**Keywords:** eurozone, monetary‐fiscal policy, insurance, transfers, COVID‐19, 欧元区, 货币‐财政政策, 保险, 转移, 新冠肺炎（COVID‐19）, eurozona, política monetario‐fiscal, seguros, transferencias, COVID‐19

## Abstract

This article argues that the incomplete economic and institutional integration of the euro area has exposed the monetary union to increasing economic divergence, which could be deepened by the COVID‐19 crisis. We discuss how monetary and fiscal measures implemented at the onset of the pandemic have contributed to mitigate the economic consequences of lockdowns, but provided limited insurance to narrow economic gaps across member countries. However, EU countries agreed on July 21, 2020 to develop, for the first time, countercyclical fiscal transfers financed by common debt issuance. We discuss the potential of this instrument to contribute to improve the resilience of the eurozone.

## 摘要

本文主张，欧元区不完整的经济一体化及制度一体化已使该货币联盟出现越来越多的经济差异，而这一差异可能因新冠肺炎（COVID‐19）危机而加深。我们探讨了大流行开始时采取的货币措施和财政措施如何促进缓解行动限制的经济结果，但在缩小成员国之间的经济差距方面提供的保障有限。尽管如此，欧盟国家于2020年7月21日首次同意通过共同发债来完成反周期财政转移。我们探讨了这一工具在促进提高欧元区复原力方面的潜力。

## Resumen

Este artículo sostiene que la integración económica e institucional incompleta de la eurozona ha expuesto a la unión monetaria a una creciente divergencia económica, que podría verse agravada por la crisis del COVID‐19. Analizamos cómo las medidas monetarias y fiscales implementadas al inicio de la pandemia han contribuido a mitigar las consecuencias económicas de los bloqueos, pero han proporcionado un seguro limitado para reducir las brechas económicas entre los países miembros. Sin embargo, los países de la UE acordaron el 21 de julio de 2020 desarrollar, por primera vez, transferencias fiscales anticíclicas financiadas por la emisión de deuda común. Discutimos el potencial de este instrumento para contribuir a mejorar la resiliencia de la eurozona..

## 1 INTRODUCTION

Only a decade after the eurozone sovereign debt crisis that almost led to the break‐up of the monetary union, the euro area is facing another threat, with the COVID‐19 pandemic leading to the most severe contraction of output ever recorded.^1^ There is now much debate about the nature and pace of the recovery that may ensue. Household consumption could continue to be subdued following the introduction of social distancing measures, higher unemployment and precautionary saving. Meanwhile, high uncertainty about possible setbacks on the health front, and the large increase in corporate debt during the lockdowns, could weigh heavily on private investment.

But another even more fundamental risk is that the current crisis will deepen the economic divergence across the Economic and Monetary Union (EMU) that started a decade ago. The severity of the recession but also the strength of the recovery could indeed differ across euro area countries for three main reasons: Some countries were affected by the pandemic earlier than others; some countries rely more on sectors (e.g., tourism) that have been heavily affected by the pandemic; and some countries have more policy space to react to the crisis. In the absence of risk‐sharing mechanisms at EU‐level, this means that the cohesion and sustainability of the monetary union could be threatened.

This article discusses how the European institutions reacted and evolved during the early stages of the COVID‐19 crisis in the first half of 2020. Member states triggered exceptional fiscal responses to address the economic and social consequences of the pandemic, but generally only proportionally to their respective fiscal capacities. The European institutions also developed emergency programs, but faced some limitations. The European Central Bank (ECB) expanded its asset purchase programs to avoid another sovereign debt crisis, but at the risk of being contested in court. The European Stability Mechanism (ESM)—created during the previous crisis—offered new credit lines to member states but with limited success. However, after a few months of difficult negotiations between member states, the unprecedented COVID‐19 crisis paved the way for a major institutional innovation, with the introduction of countercyclical fiscal transfers between countries financed by common debt issuance.

## 2 BEFORE THE COVID‐19 CRISIS: AN INCOMPLETE AND DIVERGING MONETARY UNION

The introduction of the euro in 1999 was the outcome of repeated attempts to put an end to volatile exchange rates that were considered harmful for the European Union (EU) single market. The objective of the common currency was to promote trade and economic ties between members more generally, but also to eventually foster European political integration.

However, the optimum currency area literature (Mundell, 1961) warned that a monetary union deprives member countries from independently making exchange rate adjustments to buffer country‐specific economic shocks. The resilience of a monetary union to large asymmetric shocks therefore hinges on the existence of effective risk‐sharing mechanisms that substitute foregone monetary instruments at the national level.^2^

Asdrubali et al. (1996) identify three main channels of cross‐country insurance. First, integrated *capital markets* support cross ownership of financial assets to buffer variations in domestic income. Second, individuals and public institutions can borrow or lend on *credit markets* and adjust domestic resources to temporary variations in income. And third, *fiscal transfers* across regions provide a taxpayer‐funded insurance mechanism. In practice, these channels insure up to 80% of state income shocks in the United States, with a dominant role for private risk‐sharing channels, while fiscal transfers across states only contribute residually. But in the euro area income shocks at the national level are only insured at 25% to 50% through these channels.^3^ This absence of effective cross‐country insurance mechanism exposes a monetary union to the risk of unsustainable economic divergence.

From its outset, EMU brought together countries with diverse institutional and economic conditions. The project was grounded in the faith that countries would eventually converge toward a homogeneous bloc, relying on financial market integration to mitigate the inability of participating states to autonomously adjust nominal exchange rates. However, alternative voices warned that the development of the common market could foster individual vulnerabilities, thereby driving the currency area along an unsustainable path.^4^

**Figure 1:**
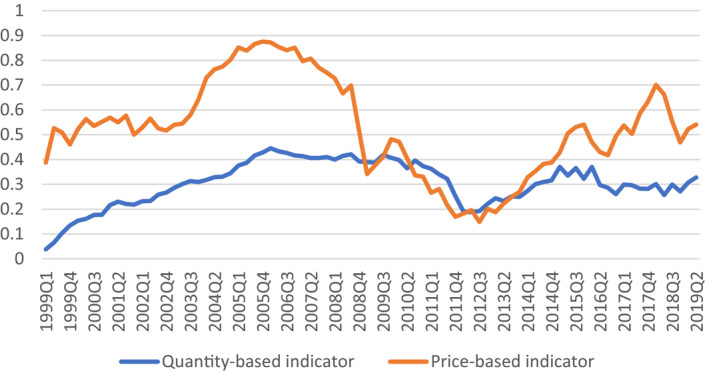
Eurozone financial integration indicators. Source: European Central Bank. Notes: The index of financial integration is based on price co‐movements of asset classes and portfolio diversification across countries. Indicators range from 0 (no integration) to 1 (full integration). Details in Hoffman et al. (2019)

Figure 1 presents the evolution of financial integration in the eurozone based on indicators of asset price co‐movements and portfolio diversification across countries.^5^ During the 2000s, financial integration was building up. Cross‐country capital flows seemed to support convergence in income per capita and provided some of the insurance required to support the cohesion of the monetary bloc. However, these developments relied mainly on short‐term interbank loans and wholesale debt markets. As documented by Merler and Pisani‐Ferry (2012), these private cross border capital flows reversed during the Great Recession (2008–2012) and led to a sudden stop in capital flows in some countries. In addition, the institutional architecture displayed a critical weakness: European banks relied heavily on national treasuries' support precisely when public finance was in distress. The urge to break this so‐called “doom loop” led the development of the EU’s banking union in 2012, but market fragmentation persisted.^6^ In the end, both *capital* and *credit market* channels of cross‐country insurance failed to buffer the shock of the financial crisis in EMU countries, while the *fiscal* channel was non‐existent.

Table 1 reports key economic indicators for selected countries accounting for roughly 80% of eurozone GDP and population. It illustrates how the financial crisis left uneven scars across the eurozone, which persisted during the 2010s, a decade of economic divergence. Peripheral countries hit by the sovereign debt crisis struggled to restore economic growth. In contrast, so‐called core countries benefited from international trade dynamism to rebound from the recession.^7^

**Table 1:** Macroeconomic indicators

	Relative GDP per capita			Unemployment rate		
	Average	Average	2020 (e)	Average	Average	2020 (e)
	2000–2008	2012–2019		2000–2008	2012–2019	
France	97	89	83	8.22	9.39	10.1
Germany	100	100	100	9.24	4.34	4.0
Italy	89	74	68	8.13	11.38	11.8
Netherlands	118	111	113	3.90	5.69	5.9
Spain	72	63	61	10.63	20.46	18.9

	General government balance			Debt to GDP ratio		
	Average	Average	2020 (e)	Average	Average	2020 (e)
	2000–2008	2012–2019		2000–2008	2012–2019	
France	−2.80	−3.55	−9.9	63.68	95.88	116.5
Germany	−2.26	0.90	−7.0	63.17	70.48	75.6
Italy	−2.98	−2.50	−11.1	106.48	133.53	158.9
Netherlands	−0.72	−0.83	−6.3	49.26	60.76	62.1
Spain	−0.21	−5.18	−10.1	45.90	96.63	115.6

The average gross domestic product (GDP) per capita during the 2010s for Italy and Spain were, respectively, at a low 74% and 63% of the German level, against 89% and 72% during the preceding decade. While Germany and the Netherlands were experiencing near full employment in 2019, France, Italy, and Spain (as well as other countries not included in the Table such as Greece and Portugal) were still confronted with relatively high levels of unemployment. Finally, even though government deficits fell relatively quickly thanks to a combination of fiscal consolidation, low interest rates, and moderate growth, debt‐to‐GDP ratios diverged widely between the two groups of countries.

The COVID‐19 pandemic is exacerbating this economic divide.Projections for 2020 (reported in Table 1) outline the increasing heterogeneity of the monetary union. This is the result of three main developments. First, as documented in this issue, from a health perspective, the impact of the pandemic was greater in Italy and Spain than in Germany or the Netherlands. Second, the direct economic cost of lockdowns is expected to be much greater in countries that rely on the services sector, and in particular on tourism, as is the case in southern Europe. Third, to tackle the economic consequences of the pandemic, countries have adopted exceptional fiscal measures.^8^ These rely on debt issuance to finance health expenses and programs to support workers and firms. In particular, temporary lay‐off benefits (such as the German “Kurzarbeit”) and bank loan guarantees have been implemented to avoid permanent lay‐offs and to protect the solvency of viable companies during the lockdowns. But the magnitude of these programs differs notably across countries.

Table 2 presents a breakdown of these policies in three main categories: direct expenses, tax deferral, and extension of guarantees to firms. Probably because countries’ fiscal spaces were not comparable at the beginning of the crisis, the magnitude of the fiscal packages (especially when excluding guarantees) appeared to be inversely proportional to the economic shock at least in the early phase of the crisis.^9^

**Table 2:** Discretionary 2020 fiscal measures

	Total	Immediate fiscal impulses	Deferral	Liquidity provisions or guarantees
France	25.80%	4.40%	8.70%	14.20%
Germany	51.90%	13.30%	7.30%	27.20%
Italy	43.90%	3.40%	13.20%	32.10%
Netherlands	15.00%	3.70%	7.90%	3.40%
Spain	12.40%	3.70%	0.80%	9.20%

**Figure 2:**
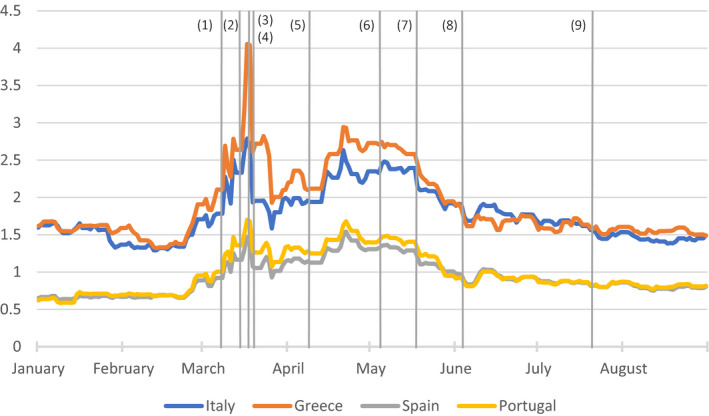
Selected Eurozone sovereign debt spread (in%). Source: Bloomberg. Spreads are reported as compared to yields on German bonds. List of events: (1) Italian nationwide lockdown. (2) German border closure. (3) ECB PEPP. (4) Suspension of the SGP. (5) ESM PCS facility. (6) German constitutional court ruling. (7) French‐German initiative for cross‐country transfers. (8) ECB PEPP increased. (9) EU Council Agreement on Next Generation EU

This divergence of situations and policies has the potential to raise three direct threats to the sustainability of EMU. First, the fact that wealthier nations can provide support to their industrial champions, while poorer countries have less resources to do so, could distort the level playing field in the single market.^10^ Second, the situation could lead to political tensions along classic divisions: a powerful narrative in southern Europe is that countries from the core benefit from the single market while lacking solidarity toward their natural trade partners, while in northern Europe it has been argued that fiscal profligacy and the lack of structural reforms are responsible for bad economic performance in the south. Finally, in the context of unprecedented economic contraction, an imperfect institutional architecture and rising public debt, the monetary union could also be exposed to sovereign debt market stress, driven by the fear of defaults or the exit of a member state. Despite their well‐known imperfections as a signaling mechanism, sovereign spreads, that is, the difference in interest rates paid by EU countries, remain a critical indicator of how financial markets assess the sustainability of the monetary union. Figure 2 presents the evolution of selected sovereign spreads in the euro area.^11^

## 3 THE LIMITS OF THE INITIAL EUROPEAN RESPONSE (MARCH‐MAY 2020)

At the beginning of the COVID‐19 crisis, European institutions and member states relied on the instruments already at their disposal to mitigate the economic contraction and their individual vulnerabilities. The objective of the decisions taken at the European level was mainly to ensure that countries could access cheap market funds to finance ballooning public expenditure. However, they were short of developing an explicit insurance mechanism. The imperfections of these first measures underline the shortfalls of the fiscal‐monetary architecture of the EU when the crisis started, as European countries had missed the opportunity to improve it during the relatively quiet times between crises.

### 3.1 An insufficient early European response on the fiscal front

Initially, it seemed that the use of the ESM was the easiest way to provide support to member states. The ESM was established in 2012 as a permanent institution to provide financial assistance to euro area countries facing market stress.^12^ ESM loans are provided with interest payments that are lower than market rates. The positive impact of such an instrument is related to the interest that is saved on newly issued debt. Access to an ESM credit facility is also a precondition to activate the ECB’s Outright Monetary Transactions (OMT) program, a cornerstone of eurozone financial stability since its announcement in 2012.^13^

The main concern with these loans is that they require countries to participate in macroeconomic adjustment programs, combining economic reforms and fiscal consolidation measures. The perceived failure and the negative social impact of austerity measures—imposed on Greece in particular—during the euro crisis in exchange for financial assistance has left deep political scars in southern European countries. Even in countries which did not participate in financial programs, for example in Italy, political discussions about the ESM can be particularly toxic.

Given the exogenous nature of the pandemic, imposing strict conditionality to mitigate moral hazard in exchange for ESM support was hardly justified this time. On April 9, 2020, euro area finance ministers agreed to develop a specific ESM credit line with limited conditionality. The newly created ESM Pandemic Crisis Support (PCS) credit facility allows countries of the euro area to borrow up to 2% of their GDP (i.e., €240 billion for the whole euro area) without the need to implement an adjustment program, as long as the money borrowed is used to finance healthcare, vaccine and treatment programs, and other crisis‐related expenses.

In addition to this new ESM credit line, euro area finance ministers also agreed to create a temporary new EU instrument, entitled “Support to mitigate Unemployment Risks in an Emergency” (SURE). This additional borrowing facility can provide up to €100 billion in loans to EU countries to fund short‐term work schemes that have been heavily relied‐upon since the beginning of COVID‐19 lockdowns in March 2020.

As for the ESM, the main advantage of SURE is that it can rely on the AAA rating of EU institutions to borrow cheaply and pass these interest savings on to member states (Claeys, 2020a). However, even if these two new instruments provide some relief to member states by allowing them to access cheap funds, they have not been perceived as improving decisively the resilience of the monetary union itself, at least in the eyes of financial markets. As shown by Delatte and Guillaume (2020), markets were even fairly disappointed by these announcements, because investors understood that providing cheap debt to fragile economies was not enough to close economic gaps in the monetary union.

Lifting contentious conditionality attached to European credit lines was a welcome first step to encourage countries to use them. However, there are two main limits to these credit lines. First, interest saved on these loans are too small to address significantly diverging vulnerabilities.Creel et al. (2020) calculated that the impact of the ESM’s PCS would, for instance, allow Italy to save 0.04% of GDP on interest rates.^14^ Second, there is a risk that participating in an ESM program would leave a negative stigma: markets could interpret an ESM request as a signal of weakness and therefore would demand higher rates when countries issue new bonds, which would then erase the savings generated by the ESM loans. Accordingly, because of these economic considerations and the political defiance towards ESM, no country has yet tapped the ESM’s PCS, while 15 countries have requested to use SURE for a total of €81.4 billion (European Commission, 2020).

Overall, the main problem of these tools is that they were not designed to solve the right problem. Access to market funds has not been a critical issue for euro area members, as demonstrated by the level of sovereign yields in recent months. This is particularly due to the decisive programs that the ECB implemented since mid‐March.

### 3.2 The crucial role of the ECB in the crisis and its limits

The ECB primary mandate is to ensure price stability in the euro zone. However, the ECB’s actions must respect two limits set down in the EU Treaties. First, the ECB is not allowed to directly finance member states or EU institutions. Second, like all EU institutions, the ECB should not act beyond its assigned competences and should only use its instruments to the extent necessary to fulfill its mandate.^15^ The ECB thus has to find the right balance to fulfill its mandate and to ensure the integrity of the monetary union while respecting the EU Treaties’ boundaries, which is not always an easy task.

In particular, two ECB instruments have been crucial to preserve the integrity of the monetary union since 2010. First, the Outright Monetary Transactions (OMT) program announced in 2012 gives the ECB the possibility to buy sovereign debt from a particular country without any limits if that country faces the risk of a self‐fulfilling liquidity crisis. However, to ensure that the ECB does not breach the prohibition of monetary financing of insolvent government, OMT is conditional on the participation in an ESM program, and requires the unanimous political approval of euro area finance ministers. Without ever being activated, the OMT program decisively insulated euro area sovereign bond markets against speculative attacks in 2012 and ultimately put an end to the euro debt crisis.^16^

**Figure 3:**
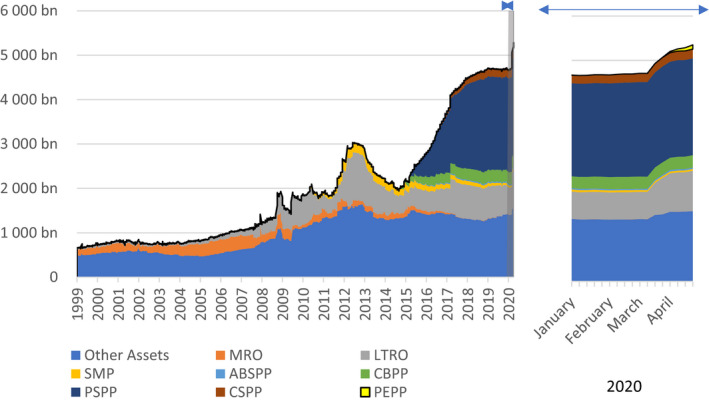
Eurosystem consolidated assets—by program (in €). Source: Claeys (2020, 2020b). The left‐hand side panel shows the evolution of the Eurosystem's balance sheet since 1999, while the right‐hand side panel zooms in on the developments since the beginning of 2020; MRO: Main Refinancing Operations, LTRO: Long‐Term Refinancing Operations (includes all types of LTROs, including VLTROs and TLTROs), SMP: Securities Market Programme, ABSPP: Asset Backed Securities Purchase Programme, CBPP: Covered Bond Purchase Programme, PSPP: Public Sector Purchase Programme, CSPP: Corporate Sector Purchase Programme, PEPP: Pandemic Emergency Purchase Programme

Second, the ECB’s Public Sector Purchase Programme (PSPP)—initiated in 2015—has allowed the ECB to continue to loosen its monetary stance significantly by acting directly on the long‐term part of the yield curve, when its main instrument—the short‐term rate—is constrained by the zero‐lower bound.^17^ The objective of this program is to bring inflation towards the ECB target, namely “below but close to 2 percent,*”* but indirectly, it also contributes to the maintenance of favorable funding conditions for member states. Therefore, to avoid breaching the prohibition of monetary financing, the ECB decided to constrain itself under some key principles. First, an issuer limit prescribes that the ECB should not buy more than a third of any country's eligible assets. Second, the distribution of asset purchases across countries follows the ECB’s capital keys, to avoid monetary policy becoming selective.^18^ As reported in Figure 3, the balance sheet of the ECB increased by a factor of three between 2007 and 2019, in large part as a result of this program.

When the COVID‐19 crisis struck, the ECB reacted very quickly to the growing economic uncertainties that was triggered by strict lockdowns, by further expanding its toolbox and by adjusting its safeguards to address the potentially asymmetric implications of the pandemic. On March 18, 2020 in particular, following a significant increase in some countries’ funding costs, the ECB announced a new asset purchase program—the Pandemic Emergency Purchase Programme (PEPP)—with an envelope of €750 billion running at least until the end of 2020. Its explicit objective was to diminish “*any risks to the smooth transmission of its monetary policy in all jurisdictions of the euro area*” (ECB, 2020). The size of the envelope and minimum end date were subsequently extended on 4 June to reflect the “*undeterre*d” commitment of the ECB to act swiftly against the deteriorating economic outlook and associated fall in inflation in the euro area.^19^ Importantly, in contrast to previous asset purchase programs, PEPP is not subject to the 33% issuer limit, and asset purchases are guided by, but are not strictly bounded by, the capital key distribution in the short term. These adjustments are meant to ensure the program is flexible enough to address specific vulnerabilities in the euro area during the pandemic.^20^ In contrast to early measures of the Eurogroup on ESM loans, financial markets reacted positively, as spreads fell quickly after the central bank's announcements (Figure 2).

Thanks to these policies, the ECB has been the main European institution providing countercyclical economic stimulus and maintaining the integrity of the monetary union over the last decade. This contradicts the optimum currency area literature which generally points to the irrelevance of monetary policy to address idiosyncratic shocks in a currency area, but the ECB managed to adapt its toolkit to make up for the lack of effective risk‐sharing mechanisms (both public and private) in the euro area. However, these programs have attracted ferocious criticisms in some countries and have even been legally challenged in Germany, with critics arguing that asset purchase programs blurred the line between monetary and fiscal policy.^21^

As a result, the Court of Justice of the EU, which was consulted by the German Constitutional Court on the legality of OMT and PSPP, considered that asset purchases are a legitimate monetary instrument as long as “*sufficient safeguards*” exist (CJEU, 2015, 2018). The Court of Justice ultimately decided that the safeguards present in these programs were compatible with the EU Treaties.

However, the situation was complicated by the ruling of the German Federal Constitutional Court on May 5, 2020, which considered that the CJEU did not appropriately assess the proportionality of the ECB’s programs. The German Court gave three months to the ECB to prove that the economic and fiscal policy effects of its programs do not outweigh its objectives, against the threat of prohibiting the participation of the German Bundesbank in the asset purchase programs of the Eurosystem. The main uncertainties raised by this ruling have been defused during the summer 2020, with a vote by the German Parliament in support of the ECB.^22^ Nevertheless, this episode underlines the critical tensions in the overlapping national and European legal environments, which could still weigh on future monetary policy decisions. Indeed, it increases the uncertainty regarding the capacity of the ECB to act decisively to dampen shocks affecting the euro area, as the central bank might find it difficult to buy assets in an unlimited fashion in the future. A strict application of the 33% issuer limit (as sought by the German Constitutional Court) could drastically constrain asset purchase programs, while a rigorous application of the capital key rule would prevent the central bank from undertaking large and persistent asymmetric interventions. By opening its balance sheet “*as much as necessary and for as long as needed*” (ECB, 2020), the ECB might have come closer to the limits of its competences.^23^

More fundamentally, the German Constitutional Court ruling highlights a vital problem of the euro area architecture: Two decades after the launch of the euro, there are still uncertainties regarding the range of instruments the ECB is allowed to use to fulfill its mandate. These ambiguities are particularly problematic because this reduces the credibility of the ECB’s policies and, in the current pandemic situation, of the PEPP. This could lead to the re‐emergence of self‐fulfilling crises in euro area sovereign bond markets, similar to what happened during the euro crisis before ECB’s OMT was announced.

In that context, the ECB repeatedly called for the development of an “*ambitious and coordinated fiscal stance*” to support the recovery, and then “*welcome[d] the European Council agreement to work towards establishing a recovery fund dedicated to dealing with this unprecedented crisis*”.^24^

## 4 TOWARDS AN EU RECOVERY FUND (MAY‐JULY 2020)

The COVID‐19 pandemic had the potential to give a critical blow to a European project that had been stagnating since the end of the last crisis. This extreme situation seemed to necessitate an innovative reaction to avoid the potential breaking apart of the common currency area, but European leaders appeared at first unable to overcome conflictual positions and to develop a decisive common solution. In particular, they could not agree on the creation of a solidarity fund, that was much‐needed to help the countries most affected by the crisis, and preferred to rely exclusively on the ECB to provide economic relief.

In that context, the Franco‐German initiative announced on May 18, 2020 came as a positive surprise, in particular after a decade of lukewarm German reception of French proposals on European integration.^25^ Indeed, the idea of common fiscal policy with joint liability had long been contentious in the EU, and any proposals for debt‐financed countercyclical transfers across countries or for the mutualization of outstanding debt were systematically discarded. In contrast, the Franco‐German proposal called for the development of a €500 billion recovery fund, that would be distributed to governments in the most affected regions in the form of transfers financed by common long‐term debt issuance. Following this initiative, the European Commission developed a proposal that was announced on May 29, 2020 for a debt‐financed €750 billion recovery fund, including €500 billion in grants and €250 billions in additional back‐to‐back loans made by the EU.

Despite stark opposition to debt‐financed transfers voiced by the so‐called “*frugal four*” governments (i.e., Austria, Denmark, the Netherlands and Sweden), the European Council^26^ reached a historical compromise on July 21, 2020, by adopting an amended proposal of the Commission's "Next Generation EU" recovery fund. This one‐off instrument will provide €750 billion to the 27 EU countries to finance targeted investments and economic reforms that, crucially, would be funded by common debt issuance. More importantly, more than half of these funds (€390 billion) are to be allocated as grants to EU countries, in effect introducing debt‐financed cross‐country transfers in the EU for the first time,^27^ while the reimbursement of the joint debt is to be serviced by the general EU budget.

Estimates of transfers by Darvas (2020) reproduced in Table 3 indicate that the allocation of grants would provide larger pay‐outs to the countries that had been worst‐hit by the COVID‐19 crisis, but would also contribute to redistributions from richer to poorer EU countries. Italy and Spain would be the largest beneficiaries in value under the terms of the program, while Bulgaria, Greece, and Croatia would be the highest beneficiaries as a share of gross national income (GNI).^28^

**Table 3:** Recovery fund: estimated allocation of grants

Top five recipients				
Absolute value transfers			% 2019 GNI	
	€ billion	% total		
Italy	84.6	22.08%	Bulgaria	9.85%
Spain	71.3	18.54%	Croatia	9.76%
France	50.1	13.18%	Greece	8.93%
Germany	47.2	12.27%	Latvia	6.52%
Poland	26.8	6.98%	Romania	6.22%

Bottom five recipients				
Absolute value transfers			% 2019 GNI	
	€ billion	% total		
Ireland	1,53	0,40%	Sweden	0,81%
Estonia	1,13	0,29%	Austria	0,79%
Cyprus	1	0,26%	Netherlands	0,79%
Malta	0,3	0,08%	Denmark	0,56%
Luxembourg	0,26	0,07%	Ireland	0,56%

The instrument is under development at the time of writing and needs to be ratified by national parliaments, so implications are still speculative at this stage. However, we can try to assess its potential to change the fundamentals of the EU’s economic architecture. On the one hand, the effects as a macroeconomic stimulus tool might be limited. Although estimated transfers are relatively large compared to the 2020 economic contraction, they are small when considering the trajectories and extent of past economic divergences. Delays in the distribution of funds and conditions associated to spending might also hinder the stimulative potential of the recovery fund.

On the other hand, European enthusiasts have celebrated the European Council agreement on the recovery fund as a "Hamiltonian moment" for Europe, that could substantially increase the stability of the EU and its monetary union. The announcement was also welcomed by financial markets with an additional fall in sovereign spreads. The analogy with the architect of U.S. fiscal federalism is attractive, since Alexander Hamilton navigated reluctant independent states towards a modest common fiscal project during exceptional political circumstances. The United States Funding Act of 1,790 mutualized war‐time debts against international trade taxes and established a free‐trade area in the country. In contrast, the current European project neither mutualizes outstanding debts, nor does it consider granting substantial taxation power to a European federal treasury at this stage (even if the term “*own resources*” for the EU is mentioned in the agreement). Overall, despite core institutional differences, the comparison might still be relevant because the EU recovery fund represents a necessary first step toward a cross country insurance mechanism.^29^

More importantly, it shows once more that a dire economic situation has fostered a sense of emergency and has renewed the overall EU political dynamics that was on hold since the last crisis. The development of common debt and explicit transfers could lead to further innovation. The large EU debt emission could contribute to the establishment of a European safe asset, and a benchmark European yield curve, and, as such could strengthen the role of the euro as a global currency. Also, the introduction of transfers could invite EU leaders to revisit the dominant role of domestic parliaments in fiscal affairs. Anchoring the facility under a suitable democratic mechanism could empower the European Parliament and could also provide the required legitimacy to monitor the effective allocation of the funds under this facility.

Overall, this initiative might not be the swift instrument to lift economies out of the recession and to restore a level playing field in the single market, but it still represents a critical commitment to economic integration and cross‐country solidarity in the EU.

## 5 CONCLUDING REMARKS

At the time of writing, many uncertainties remain regarding the development of the pandemic and its economic implications. But, once again, Europe is facing an existential challenge. As an echo of the prophecy of Jean Monnet that “*Europe will be forged in crisis,*” the COVID‐19 crisis has raised the perception of emergency and has convinced European leaders that the prevalent economic and institutional architecture was not sustainable.

The recovery fund represents an additional step to assert the irreversibility of the EMU and to release the ECB from its excessive policy burden. The implementation and development of this initiative will reveal whether it is a foundational Hamiltonian moment or just a transactional and time‐limited Marshall plan.

## ENDNOTES

1 In its September Economic Outlook report, the OECD (2020) expects GDP in the euro area to fall by 7.9 percent in 2020.

2 A more complete consideration of the Optimum Currency Area literature is presented in De Grauwe (2020).

3 See European Commission (2016) for detailed estimates.

4 For a comprehensive discussion, see Frankel and Rose (1998).

5 Computations here are from ECB. For a presentation of these indicators, see Hoffman, Kremer and Zaharia (2019).

6 Schnabel and Véron (2018) discuss different scenarios to complete the common banking supervisory environment that was initiated in 2012. Critically, it depends on the objective and at a minimum, the bank‐sovereign vicious cycle should be severed. A more ambitious proposal would promote a unified euro area banking market to foster an efficient allocation of funds across the bloc.

7 The ‘core’ versus. ‘periphery’ classification dates back at least to Bayoumi and Eichengreen (1993), who identify statistical characteristics to sort EMU countries into two groups. During the sovereign debt crisis, the terminology was used to distinguish countries under market stress (e.g. Greece, Ireland, Portugal, Spain, Italy) from another group that was relatively spared by these adverse market developments (e.g. Germany, France, Belgium, the Netherlands, Luxembourg, Austria, etc.).

8 On March 20, 2020, the European Commission activated the general escape clause of the Stability and Growth Pact, effectively suspending the EU’s fiscal rules, and allowing countries to rely massively on debt issuance to finance these programmes. On 19 March, the Commission had already relaxed EU State aid rules in order to allow member states to support businesses.

9 Guarantees differ from deficit and delayed tax collection in the sense that their effect is essentially to maintain access to funding for firms rather than by providing direct support.

10 Controversial rescue packages for grounded airline companies are illustrative of this point, because government bailouts raise concerns of the threat of distortion to competition. In May 2020, the European Commission initially contested a bailout plan for Lufthansa by the German government, precisely because domestic policy‐makers had incentives to strengthen airline market power. See Motta and Peitz (2020) for a complete discussion of these issues.

11 For a detailed analysis of the reaction of sovereign spreads to European decisions during the first months of 2020, see Delatte and Guillaume (2020).

12 The ESM replaced the temporary European Financial Stability Facility (EFSF), and complemented the relatively small EU‐based programme, the European Financial Stabilisation Mechanism (EFSM), both created in 2010.

13 We discuss ECB programmes, and especially OMT, in more detail in Section 2.2.

14 Together with SURE, and the European Investment Bank (EIB)’s pan‐European guarantee fund (another European initiative aimed at increasing the firepower of the EIB during the crisis that was announced at the same time by the Eurogroup), total interest savings for Italy could be equivalent to only 0.08% of its GDP.

15 The first principle is specified in the Article 123 of the Treaty of the European Union. The second is related to the principles of proportionality and subsidiarity, as stated in the Article 5 of the same treaty.

16 See Camous and Cooper (2019) for a formal analysis of the effectiveness of OMT programme.

17 Technically, the zero lower bound arises because of the issuance of paper currency, which effectively guarantee a zero nominal interest rate and hence a floor on interest rate cut. There is an intense debate about the effectiveness of conventional and unconventional monetary policies at the zero‐lower bound, but generally, central banks have hesitated to rely on large negative interest rate cuts.

18 The ECB capital key is determined in proportion to a country's population and GDP.

19 “*Undeterred*” is the expression chosen by the President of the ECB Christine Lagarde in May 2020 to convey the determination of the institution to act decisively to preserve the integrity of the monetary union.

20 In addition to the PEPP, the ECB announced a package of measures to ease its policy. See Claeys (2020, 2020b) for the list of measures since the start of the COVID‐19 crisis.

21 Critics also attacked these programmes for not being supportive enough, with some suggesting that too stringent criteria for liquidity provision by the ECB could push countries or banks into unnecessary insolvency. See Claeys and Goncalves Reposo (2018) for a presentation of the shortfalls of ECB collateral framework and Götz et al. (2015) for a discussion of the Emergency Lending Assistance for banks.

22 The institutional management of this German crisis involved the Bundesbank providing Eurosystem documents justifying the proportionality of the ECB’s policies to the German Ministry of Finance and to the Bundestag, which then voted a motion supporting the ECB. For a detailed and critical appreciation of the ruling, see Bofinger et al. (2020).

23 At the end of August 2020, the ECB has engaged 37% of the € 1,35 trillion PEPP envelope in asset purchase, with substantial deviations from the capital key.

24 ECB, introductory statement to the press conference, April 2020, available at: https://www.ecb.europa.eu/press/pressconf/2020/html/ecb.is200430~ab3058e07f.en.html

25 On September 26, 2017, the newly elected French President Macron presented his roadmap for future European integration at the Sorbonne University in Paris, suggesting to lift "*red lines*" and open "*new horizons*". It included the development of a stabilization fund to promote common investment and an insurance against idiosyncratic shocks. In contrast, a view often expressed by German policy‐makers is that a political union with democratic oversight over transfers should be a pre‐condition to enable cross‐country transfers. See https://www.diplomatie.gouv.fr/en/french‐foreign‐policy/europe/news/article/president‐macron‐s‐initiative‐for‐europe‐a‐sovereign‐united‐democratic‐europe for the text of the speech.

26 The European Council was created in 1974 as an informal forum for European Union heads of state or government. It was formalized as an institution in 2009 under the Treaty of Lisbon, endowed with a permanent presidency. It decides on broad political directions and priorities for the European Union, generally at the qualified majority, but specific topics require the unanimous agreement of participants. The latter applies to decisions related to EU resources and indirect taxation.

27 Common debt issuance in itself is not an innovation: Horn, Meyer and Trebesch (2020) document dozen of EU debt issuance since the 1970s in order to do back‐to‐back loans guaranteed by member states and distributed to countries in crisis.

28 This is in gross terms, net benefits by individual countries are much more difficult to predict and will depend on a multitude of factors, including future growth and demand spillovers across countries. The historically low level of interest rates considerably reduces the risk that these transfers do not profit all EU countries.

29 Note also that the contribution of Alexander Hamilton to the overall federal economic architecture of the U.S. is limited, since it took another century to develop centralized monetary and banking supervisory powers. For a historical perspective on U.S. and EU institutional developments, see for instance the introduction to Thomas Sargent (2012) Nobel lecture or Henning and Kessler (2012).
